# Effect of Diffusion Annealing Temperature on the Formation Process and Properties of a Carbon–Aluminum Composite Layer on Pure Titanium

**DOI:** 10.3390/ma12121994

**Published:** 2019-06-21

**Authors:** Hongzhan Li, Youping Ma, Zhengxian Li, Shouchang Ji, Yanfeng Wang, Yifei Wang

**Affiliations:** 1 School of Metallurgical Engineering, Xi'an University of Architecture and Technology, Xi'an 710055, China; 2 Corrosion and Protection Center, Northwest Institute for Nonferrous Metal Research, Xi’an 710016, China; lzxqy725@163.com (Z.L.); luckystar_Ji@163.com (S.J.); yfwang420@163.com (Y.W.); wyf_524@sina.com (Y.W.)

**Keywords:** titanium, carbon–aluminum composite layer, wear resistance

## Abstract

A carbon–aluminum composite layer was prepared on the surface of pure titanium by double glow plasma carburizing, magnetron sputtering aluminizing, and vacuum-diffusional annealing treatment. The microstructure, phase composition, and properties of the composite layer obtained at different annealing temperatures were investigated by scanning electron microscopy, energy-dispersive X-ray spectroscopy, X-ray diffraction, X-ray photoelectron spectroscopy, and the ball-on-disc wear method. Results showed that the layer contained a mixture of TiAl_3_, Ti_2_Al_5_, and TiC phases at 650 °C for 6 h, which can significantly enhance the hardness and wear resistance of pure titanium. The layer exhibited a higher hardness of around 1231 HV_0.1_, a lower friction coefficient of 0.33, and lower wear volumes of 0.018 mm^3^ than those of the titanium substrate.

## 1. Introduction

Titanium and its alloys are widely used in the aerospace, marine, machinery, chemical, biomedical, and petroleum industries due to their low density, high strength, and excellent corrosion resistance [1,2]. However, it is difficult for titanium and its alloys to be applied under severe wear and friction conditions because of their low surface hardness, high coefficient of friction, and high tendency to adhesive wear. In most cases, the shortcoming of poor wear resistance severely affects the wide application of titanium alloys [3,4,5]. Therefore, the application of surface treatment technology to improve the wear resistance of titanium and its alloys has become an effective way to solve this problem [6].

Until now, many surface treatment techniques have been investigated to enhance the wear resistance of titanium and its alloys, such as plasma electrolytic oxidation (PEO) [7,8], laser alloying [9,10,11], high-velocity oxygen fuel (HVOF) [12], physical vapor deposition (PVD) [13,14], chemical vapor deposition (CVD) [15], carburizing [16,17,18], and aluminizing [19,20,21]. Khorasanian et al. [7] and Aliasghari et al. [8] found that the PEO coating consisted of rutile and anatase and the non-stoichiometric TiO oxide, which had a very dense structure with no significant crack or porosity, improved the wear resistance of the substrate. Laser alloying technology is an effective process for improving wear resistance, which is performed by melting the surface of a substrate with additional materials, mixing the components together, and rapidly solidifying the mixture [9]. Some researchers have prepared Ti–B coating and Ti–Al intermetallic coating on the surface of titanium alloys by laser alloying technology, which can significantly improve their wear resistance [10,11]. Utua et al. [12] studied the wear resistance of Al_2_O_3_–TiO_2_ coating deposited on titanium by HVOF then re-melted by electron beam. The results show that the sliding wear resistance and the hardness of the coating were significantly higher in comparison with that of the titanium substrate.

Chen et al. [13] designed and prepared a TiN/ZrN (ceramic/ceramic) nanoscale multilayer coating on the Ti6Al4V titanium alloy surface by the physical vapor deposition (PVD) process. The results show that the erosion resistance rate of the Ti6Al4V titanium alloy with the TiN/ZrN coating is 15.5 times higher than that of the uncoated one because of its microstructure, higher hardness, and elastic modulus. Vadiraj et al. [14] investigated the fretting wear resistance of TiN coating prepared by Physical Vapour Deposition (PVD), and the PVD TiN coating has shown the best fretting wear resistance with minimum friction coefficient, less wear scar depth and diameter, and minimum wear rate compared to other coatings of ion implanted and thermally oxidised. Sitek et al. [15] studied the microstructure, phase composition, and properties of the titanium aluminide diffusion layers produced on Ti6Al4V by Chemical Vapour Deposition (CVD), and the layers show high hardness of about 800 HV_0.05_ after CVD aluminizing and good adhesion to the substrate. Typically, the majority of these methods can significantly improve the hardness and wear resistance of titanium and its alloys. However, some of these methods have the disadvantage of low bonding strength between the coating and substrate. Carburizing and aluminizing have been widely studied and applied due to the excellent bonding strength, high hardness, and great wear resistance of the diffusion layers.

Belkin et al. [16] investigated the influence of electrolyte compositions on the properties and structure of commercial pure titanium (CP–Ti) in the process of anode plasma electrolytic carburizing (PEC). The studies indicated that the anode PEC of the CP–Ti samples led to the formation of a solid solution of carbon in titanium, and the friction coefficient ranged from 0.29 ± 0.01 to 0.10 ± 0.01. Zhao et al. [17] studied the contact solid carburization method to fabricate TiC coatings on a titanium alloy and concluded that the coating was composed of equiaxed TiC grains and was completely dense (no porosity). The coatings exhibited high hardness and excellent coating–substrate adhesion strength. Afarani et al. [18] synthesized a Ti(C, N) ceramic layer on the surface of a Ti–6Al–4V alloy by the pack nitro-carburizing method and showed that the corrosion resistance of the alloy was improved dramatically. Rastkar et al. [19] fabricated a surface layer containing TiAl_3_ and TiAl_2_ with plasma pack aluminizing where the thickness of the surface layers was up to 300 μm and the hardness of the surface layers was up to 600 HV_0.1_. Zhang et al. [20] studied the effect of hot-dip aluminizing and interdiffusion treatment on a Ti–6Al–4V alloy. They concluded that a TiAl_3_-rich coating was fabricated on the surface of the Ti–6Al–4V alloy, which improved the oxidation resistance. Szkliniarz et al. [21] investigated the oxidation resistance of a Ti–47Al–2W–0.5Si alloy modified by the aluminizing method. They showed that the generated hermetic and compact aluminum diffusion coating mainly consisted of a TiAl_2_ phase, which had a significant effect on the improvement of cyclic oxidation resistance in the case of the investigated alloy.

Most investigations have shown that a single method of carburizing or aluminizing on the surface of titanium and its alloys can improve the hardness and wear resistance. Until now, there have been no reports on the treatment of a carbon–aluminum composite layer on a titanium alloy. Therefore, in this paper, a new idea of carbon–aluminum composite layer on pure titanium was proposed. First, double glow plasma carburizing was carried out on a pure titanium substrate to diffuse carbon from high-purity graphite into the substrate to form a TiC layer. Then, the aluminum layer was formed on the surface of the carburized layer by magnetron sputtering and, finally, the carbon–aluminum composite layer was formed by diffusion heat treatment in a vacuum furnace. According to the experimental results, the microstructure, composition distribution, phase structure, surface composition, and wear resistance of the carbon–aluminum composite layer were analyzed.

## 2. Materials and Methods 

Plates of commercially pure TA2 titanium (Fe ≤ 0.30 wt.%, C ≤ 0.10 wt.%, N ≤ 0.05 wt.%, H ≤ 0.015 wt.%, O ≤ 0.25 wt.%, balance Ti) with dimensions of 20 mm × 20 mm × 2 mm were used as the substrates in this study and were pretreated with polishing, cleaning, and drying.

**Figure 1:**
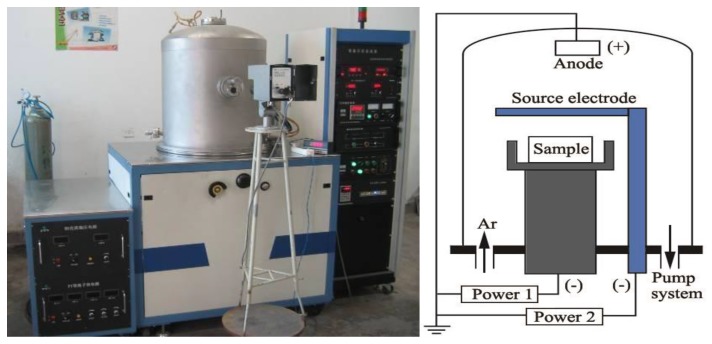
Schematic of the double glow plasma carburizing system and the equipment.

The carburized layer of commercially pure TA2 titanium was pretreated by the double glow plasma surface metallurgy technique [22]. Figure 1 shows a schematic of the double glow plasma carburizing system and the equipment. The sample was fixed on the work electrode and the source electrode was made of high-purity graphite. In an Ar plasma atmosphere, the carbon atoms and ions were sputtered by the glow discharge between the anode and source electrode and were enhanced further by the second glow discharge between the anode and work electrode; the second glow discharge could heat the sample to supply more energy for the carbon diffusion. The processing parameters are displayed in Table 1.

**Figure 2:**
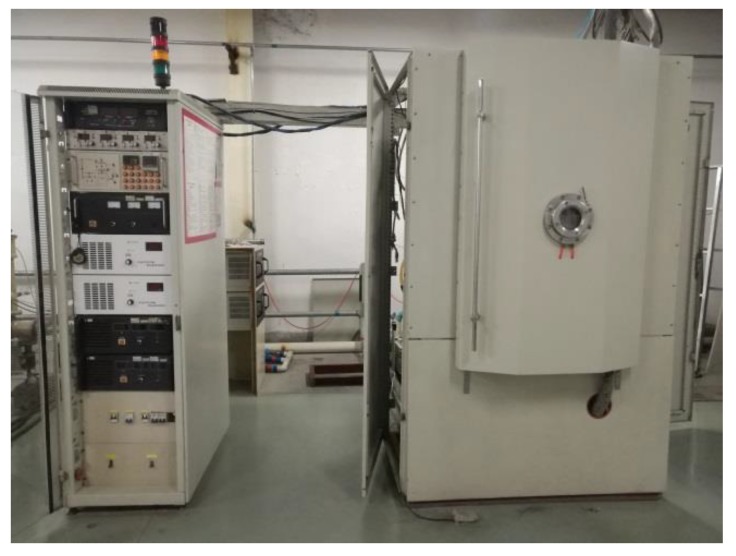
The magnetron sputtering equipment.

The loose carbon layer on the surface of the carburized specimen had been removed previously when the Al layer was prepared by magnetron sputtering using a single target of pure Al (99.99%). The Al layer was obtained as a source for the carbon–aluminum composite layer. Figure 2 shows the magnetron sputtering equipment (SP-0810AS, Beijing Powertech Technology Co., Ltd, Beijing, China). The processing parameters are displayed in Table 2.

**Figure 3:**
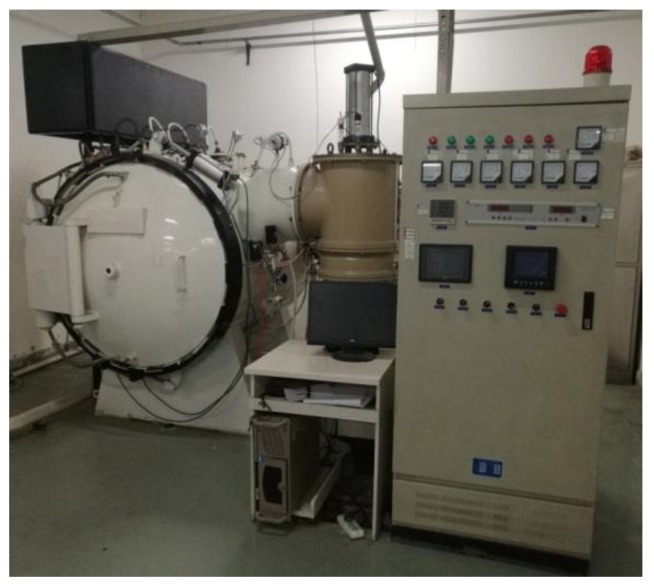
Vacuum heat treatment furnace.

The carbon–aluminum composite layer was prepared by a vacuum-diffusional annealing treatment using vacuum heat treatment furnace (ZR-80-9W3, Xi'an Qinfei Electric Furnace Co., Ltd Xi’an, China) (Figure 3), during which both the heating and cooling rates were 5 °C/min. Four samples were prepared under each process condition for field-emission scanning electron microscopy, X-ray diffraction, hardness, and wear properties testing, respectively. The processing parameters are displayed in Table 3.

The microstructure of the carburized sample was observed by an inverted metallurgic microscope (Axio Vert. A1, Carl Zeiss AG, Jena, Germany). The morphologies and elemental microanalysis of the samples were observed by field-emission scanning electron microscopy (SEM, JSM-6460F, JEOL, Tokyo, Japan) operated at 20 kV, and an energy dispersive X-ray spectrometer (EDS, Oxford INCA, Oxford, UK) detector, respectively. The Al layer on the surface of the samples was removed in a solution of 10 wt.% NaOH for the XPS, XRD, micro-hardness, and friction coefficient examination. X-ray photoelectron spectroscopy (XPS, ESCALAB 250Xi, Thermo Fisher Scientific, Waltham, MA, USA) apparatus was used to analyze the composition of the carbon–aluminum composite layer with Al Ka (1486.6 eV) and a pass energy of 100 eV. Before measuring, the samples were bombarded with 2keV Ar ion for 1 min to clean their surfaces. The phase structure of the samples were characterized by X-ray diffraction (XRD, D/max 2200 PC, RIGAKU, Tokyo, Japan) with Cu Kα radiation, a tube voltage of 40 kV, a tube current of 40 mA, a range of 2θ from 10° to 90°, and a scanning speed of 8°/min.

The micro-hardness test of the sample was carried out in a micro-hardness tester (HXD-1000 TMSC/LCD, Shanghai Optical Instrument Plant, Shanghai, China), with a 100 g of load, and 15 s of dwell time. The wear resistance and friction coefficient of the sample were tested using a friction and wear tester (MS-T3000, Lanzhou Institute of Chemical Physics, Lanzhou, China) under a dry sliding condition at room temperature. The counterpart was a silicon nitride ball that was 5 mm in diameter. The samples were rotated at a rate of 500 r/min with a load of 100 g for 30 min, and the rotating radius was 3 mm. When the wear tests were completed, the cross-sectional geometry of the wear scars was analyzed by a profilometer (MFT-4000, Lanzhou Institute of Chemical Physics, Lanzhou, China), and the wear volumes were calculated using the data of the geometric profile. The wear volumes were calculated using the following equation [23]:

V_W_ = [t(3t^2^ + 4b^2^)2πr]/6b, (1)

where Vw is the wear volume (mm^3^), t is the wear scar depth (mm), b is the wear scar width (mm), and r is the wear track radius (mm). For comparison, pure Ti samples were also tested under the same conditions.

## 3. Results

### 3.1. Microstructure of the Carburized Layer

**Figure 4:**
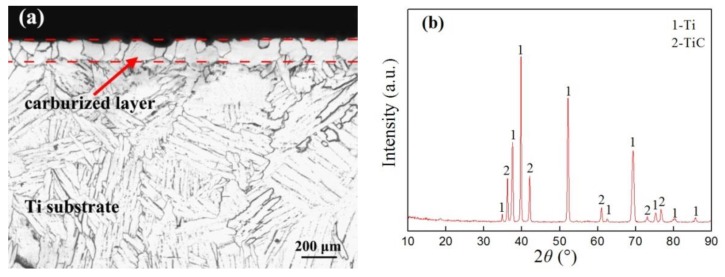
Cross-sectional metallographic image (**a**) and X-ray diffraction (XRD) pattern (**b**) of the carburized layer on the pure Ti substrate.

From the metallographic structure shown in Figure 4a, it can be seen that a carburized layer was formed with an obvious boundary on the surface of the commercially pure TA2 titanium using double glow plasma carburizing. The thickness of the carburized layer was about 100 μm, which is a dense layer of equiaxed grain structure. The corresponding XRD pattern is shown in Figure 4b, where the carburized layer mainly consists of the Ti phase and TiC phase. Guo et al. [24] reported that the higher hardness TiC phase and dissociated state of carbon were formed in the carburized layer by double glow plasma carburizing on the surface of the pure titanium. Nishimoto et al. [25] found that the carburized layer mainly consisted of an α-Ti phase and TiC phase by the spark plasma sintering method. The diffraction peaks at 2θ = 37.9°, 39.8°, and 52.6° corresponded to Ti, and the peaks of TiC were detected at 2θ = 36.2°, 42.1°, and 60.9°. The diffraction peaks of Ti shifted to the left when compared with the standard card, which may be related to the fact that the carbon solid solution distorted the lattice structure, resulting in the shifted diffraction peaks in the XRD. This finding is in accordance with the result reported by Xing et al. [26], where the carburized samples showed that peaks of the α-Ti phase had shifted slightly to lower angles. 

### 3.2. Composition of the Carbon–Aluminum Composite Layer

**Figure 5:**
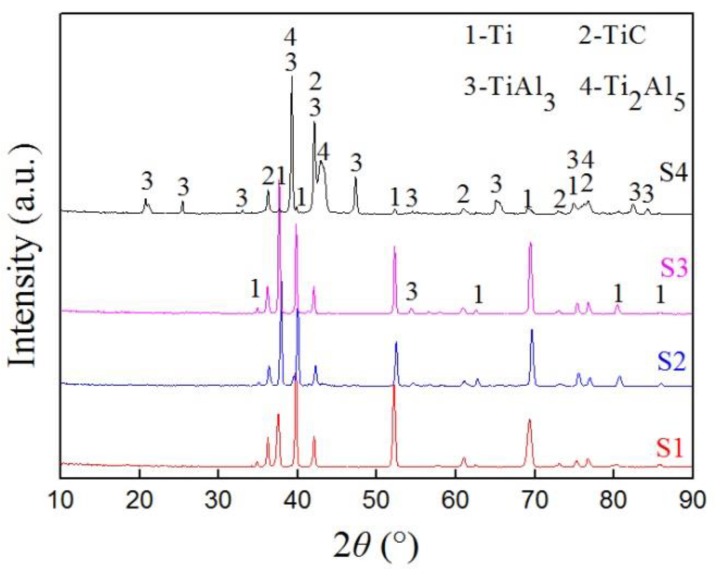
The XRD patterns of samples S1–S4.

The X-ray diffraction patterns for samples S1–S4 are given in Figure 5. The results show that the intermetallic phases of TiAl_3_ and Ti_2_Al_5_ formed gradually during the process of the vacuum-diffusional annealing treatment. Similar to the diffraction peaks of the carburized sample (Figure 4b), the XRD pattern of samples S1–S4 showed that the diffraction peaks of Ti shifted to the left. Compared with the XRD pattern of sample S1, it can be seen that the intensity of the diffraction peaks of TiAl_3_ were noticeably increased, while the peaks of TiC did not change significantly (Figure 5, S2 and S3). Combined with the different vacuum-diffusional annealing temperature of samples S2–S4, it is apparent that the TiAl_3_ phase is formed gradually with the increase of temperature. It was noticeable that the diffraction peaks at 2θ = 39.2°, 42.1°, and 47.3° corresponded to TiAl_3_, and the peaks at 2θ = 39.2°, 43.3°, and 76.7° corresponded to Ti_2_Al_5_. Meanwhile, two of the Ti_2_Al_5_ peaks overlapped the peak of TiAl_3_ at 2θ = 39.2° and the peak of TiC at 2θ = 76.7°, respectively (Figure 5, S4). The XRD patterns indicate that the carbon–aluminum composite layer can be obtained on pure titanium and contains the TiC, TiAl_3_, and Ti_2_Al_5_ phases. 

**Figure 6:**
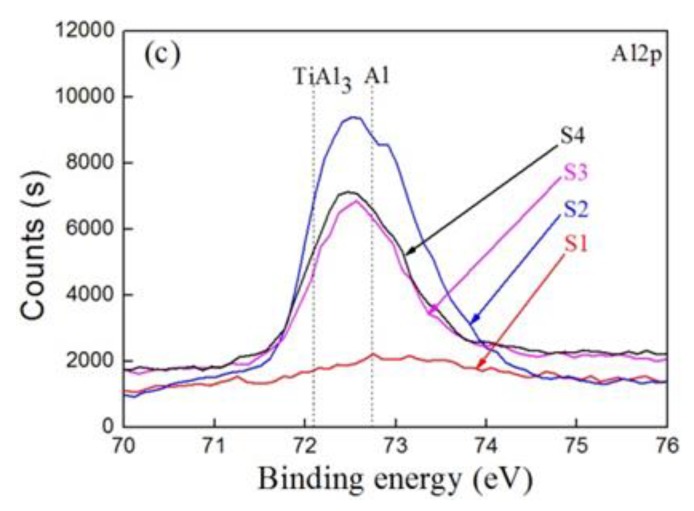

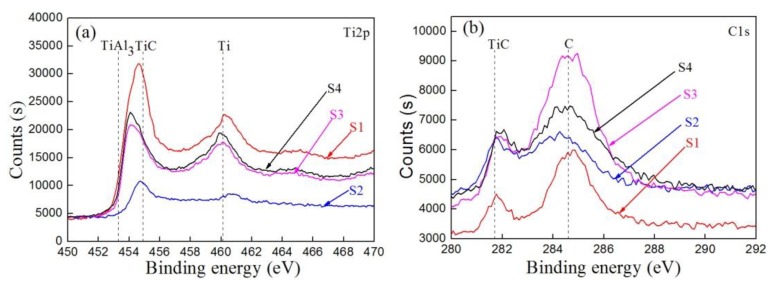
X-ray photoelectron spectroscopy of samples S1–S4: (**a**) Ti2p, (**b**) C1s, (**c**) Al2p.

Figure 6a–c presents the XPS spectra of Ti2p, C1s, and Al2p for the S1–S4 samples. For the Ti2p spectra of sample S1 in Figure 6a, the peaks at the binding energies 460.4 eV and 454.6 eV are the typical binding energies for Ti and TiC. The peaks at 460.7 eV and 454.6 eV of S2 also corresponded to Ti and TiC. The characteristic peak at the binding energy of 460.1 eV (S3) and 459.8 eV (S4) can be divided into two peaks for TiAl_3_ and TiC, separately. Figure 6b shows the C1s spectra of the samples, where one of the peaks at 281.9 ± 0.2 eV (S1–S4) corresponded to TiC, and the other at 284.6 ± 0.2 eV (S1–S4) was most likely related to C (graphite). These results are also consistent with the investigations by Felten et al. [27] and Li et al. [28]. As shown in Figure 6c, in the Al2p spectra of the S2–S4 samples, the peaks at a binding energy of 72.5 ± 0.1 eV may be separated into TiAl_3_ and Al, and the weak peak of S1 can be ignored. Based on the XPS measurements analyzed above, it can be concluded that the carbon–aluminum composite layer consisted mainly of TiC and Ti–Al phases. 

### 3.3. Cross-Sectional Microstructure

**Figure 7:**
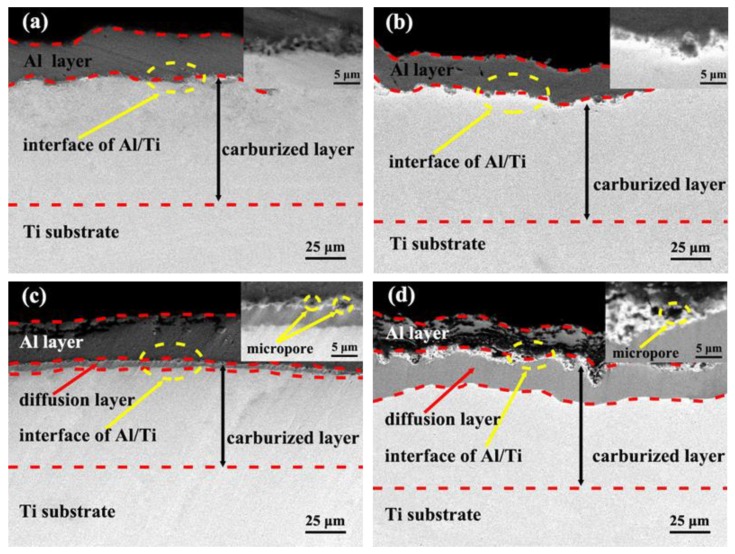
The cross-sectional back-scattered electrons (BSE) micrographs of the samples: **(a)** S1, **(b)** S2, **(c)** S3, **(d)** S4.

The cross-sectional back-scattered electrons (BSE) micrographs in Figure 7 show the formation process of the carbon–aluminum composite layer on pure titanium with different parameters of the vacuum-diffusional annealing treatment. It is known from the principle of BSE that different colors in the picture present the variation of atomic number and that the color becomes lighter with an increasing atomic number. It was observed that the Al atoms diffused into the carburized Ti substrate after the annealing experiments conducted at various temperatures of 550, 600, and 650 °C, resulting in the formation of a diffusion layer. The gray and white regions shown in Figure 7a on each micrograph correspond to the Al layer and carburized Ti substrate, respectively. The atoms of carbon as a solid solution in the carburized Ti substrate were unclear. It can be seen that there was a gray/white interface zone in the micro-area of the interface, which may be the diffusion layer (Figure 7a) of Al atoms and Ti atoms that have diffused into each other, which formed during the cyclic bombardment process of magnetron sputtering aluminizing. The cross-sectional micrographs (Figure 7b) are almost the same as those in Figure 7a, where the diffusion layer was not significantly formed. This indicated that the Al atoms diffused into the carburized Ti substrate at 550 °C more slowly. The cross-sectional micrographs presented in Figure 7c,d show that the diffusion layers formed with an average thickness of about 5 µm and 21 µm, and the micropores formed close to the Al/Ti interface by increasing the vacuum-diffusional annealing temperatures to 600 °C and 650 °C. According to the Kirkendall effect [29], the formation of micropores can be related to the higher diffusion coefficient of Al atoms in the carburized Ti substrate than that of the Ti atoms. 

**Figure 8:**
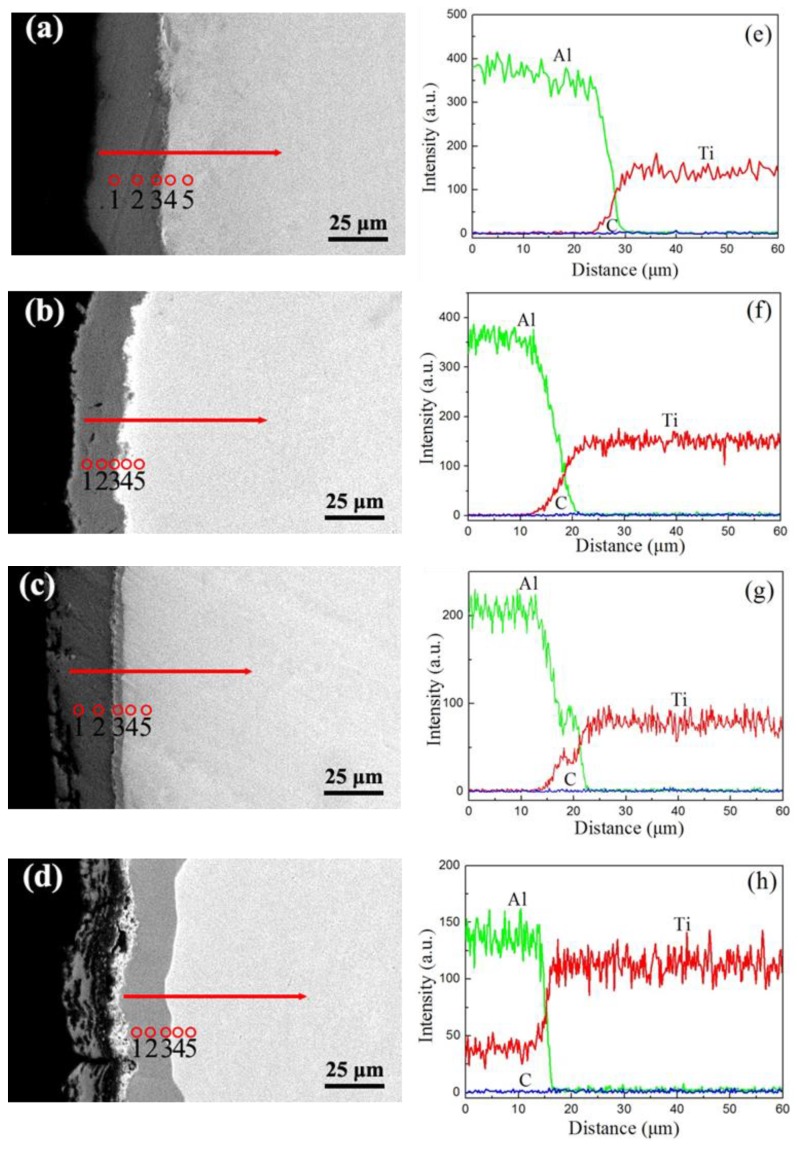
The energy-dispersive X-ray spectroscopy linear scanning and point scanning analysis of the samples: (**a**,**e**) S1, (**b**,**f**) S2, (**c**,**g**) S3, (**d**,**h**) S4.

Figure 8 shows the corresponding linear scanning and point scanning of the EDS spectrum and Table 4 lists the results of the EDS quantitative analysis in positions 1–5 in Figure 8a,e–d,h. As seen in Figure 8a,e, Ti and Al diffused into each other in the process of magnetron sputtering aluminizing and an interdiffusion region was formed on the Al/Ti interface with an average thickness of about 6 µm. The movement of atoms from Al toward Ti was about 3 μm. It should be noted that the content of Al declined gradually from position 1 to position 4 (Table 4, S1), which confirmed that the Al atoms from the surface carburization layer diffused into the Ti substrate during the magnetron sputtering aluminizing. The content of carbon was not significantly changed in the Al layer.

With an increase in the vacuum annealing temperature, the interdiffusion region of Al and Ti increased to 8 µm at 550 °C for 6 h (Figure 8b,f), and the Al atoms diffused into Ti substrate about 4 µm in depth. The thickness of the interdiffusion region of Al and Ti increased to 10 µm, and the diffusion layer reaches about 5 μm at 600 °C for 6 h (Figure 8c,g). The results showed that significant mutual diffusion of Ti atoms and Al atoms occurred at the Al/Ti interface (Table 4, S2 and S3). Figure 8d,h indicate that the thickness of the diffusion layer was 21 µm at 650 °C for 6 h. In Table 4, for S4, the content of carbon in this region in positions 1–5 exhibits a gradient change due to the double glow plasma carburizing. Under this condition, it is clear that the composite layer mainly consisted of TiC and Ti-Al phases, which formed due to the diffusion of Al into the carburized layer. According to the principle of thermal diffusion, the diffusion coefficient increases with the increase of temperature. In this paper, the higher the temperature, the higher the diffusion rate of the Al atoms. In addition, the diffusive depth increased at the same time; therefore, the thickness of the diffusion layer increased. 

### 3.4. Hardness 

**Figure 9:**
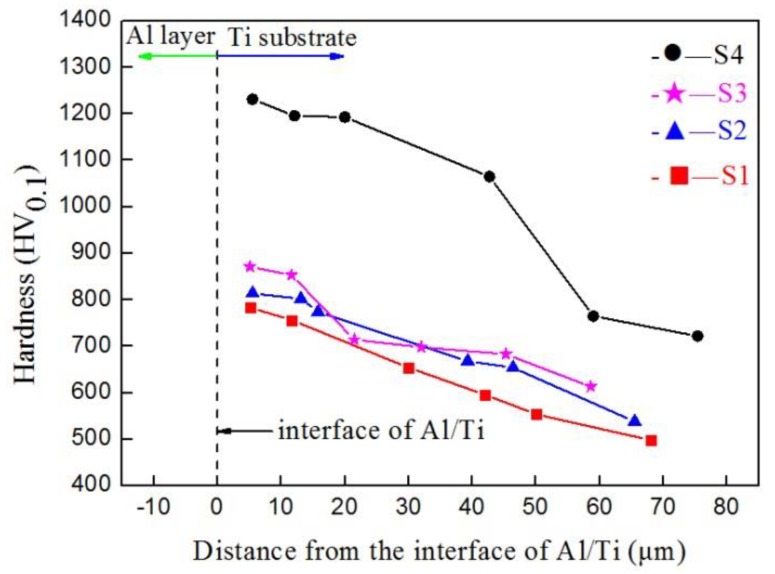
Hardness distribution on the cross-section of samples S1–S4.

Figure 9 shows the hardness distribution of the cross-section of the samples. It can be seen that the hardness of the S1–S3 samples showed essentially the same trends, and the highest hardness was between 783 HV_0.1_ and 871 HV_0.1_, where the hardness obviously decreased with the increase in distance. The hardness of sample S4 reached 1231 HV_0.1_ at 6 μm; with an increasing distance, the hardness still reached 720 HV_0.1_ at 75 μm. Compared with samples S1–S3, the hardness of sample S4 greatly improved, which may be related to the large number of Ti–Al phases formed in the carbon–aluminum composite layer. This will be discussed later in Section 4.

### 3.5. Friction and Wear Properties

**Figure 10:**
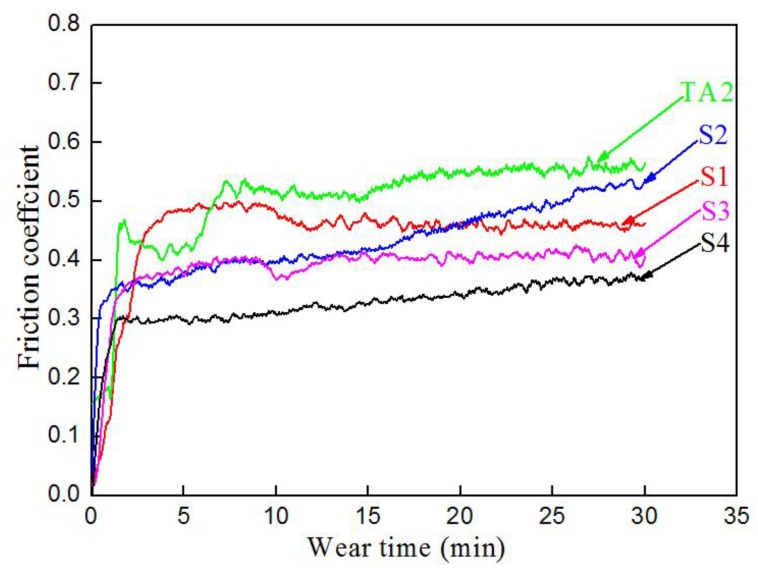
Friction coefficient of samples S1–S4 and TA2.

Figure 10 shows the friction coefficient of samples S1–S4 and TA2 at room temperature for 30 min. It is clear that the friction coefficient curves increased dramatically in the first few minutes and became relatively stable after 3 min, except for TA2. The average values of the friction coefficients of TA2 and the samples were 0.53 (TA2), 0.47 (S1), 0.45 (S2), 0.39 (S3), and 0.33 (S4), respectively. The sample S4 exhibits a low friction coefficient compared to the friction coefficient of the samples S2, S3, and TA2. 

**Figure 11:**
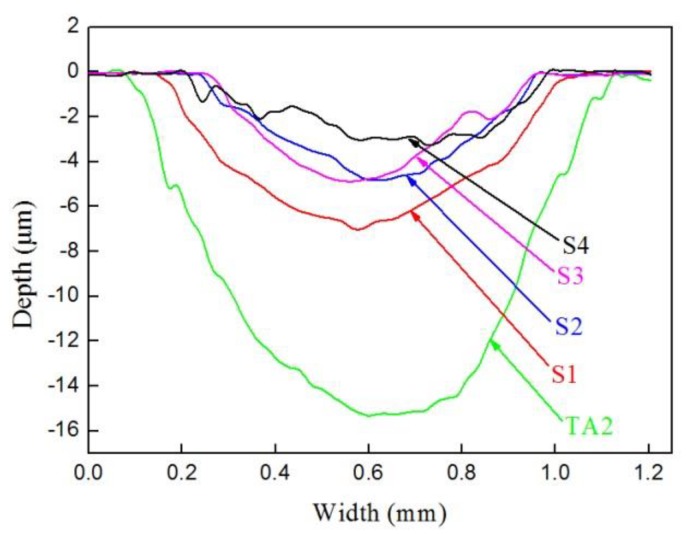
Wear scar profile curves of samples S1–S4 and TA2.

**Figure 12:**
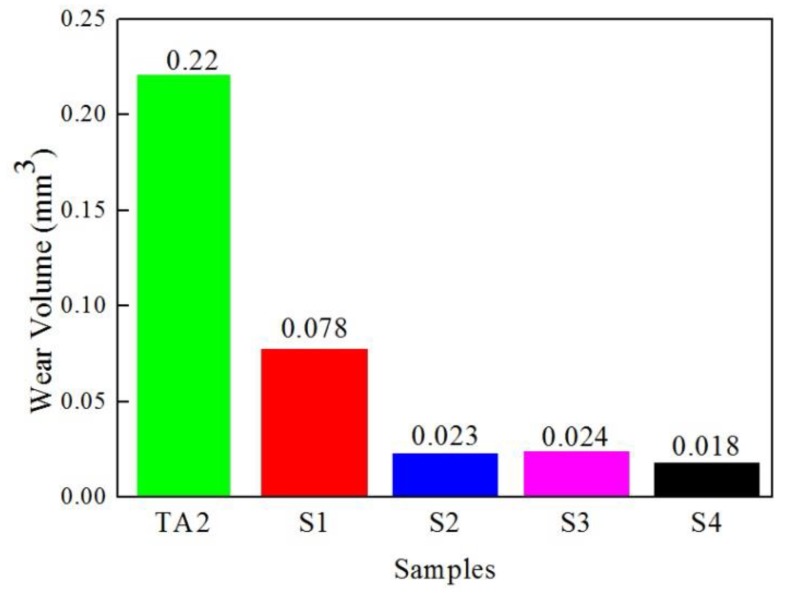
Wear volumes of samples S1–S4 and TA2.

Figure 11 shows the wear scar profile curves of samples S1–S4 and TA2 measured by a profilometer. Compared with samples S1–S4, it was found that the wear scar depth and width of TA2 were deeper and wider. The values of the wear scar depth and width became smaller and smaller from S1 to S4, while the wear scar depth and width of S2 and S3 were almost the same. Table 5 lists the geometry parameters of the wear scars profile measured by the profilometer and the wear volumes calculated by Equation (1). From Figure 12, it is evident that the wear volumes of TA2 and the samples were 0.22 mm^3^ (TA2), 0.078 mm^3^ (S1), 0.023 mm^3^ (S2), 0.024 mm^3^ (S3), and 0.018 mm^3^ (S4), respectively, which indicates that S4 had the best wear resistance.

## 4. Discussion

### 4.1. The Formation Process of the Carbon–Aluminum Composite Layer

**Figure 13:**
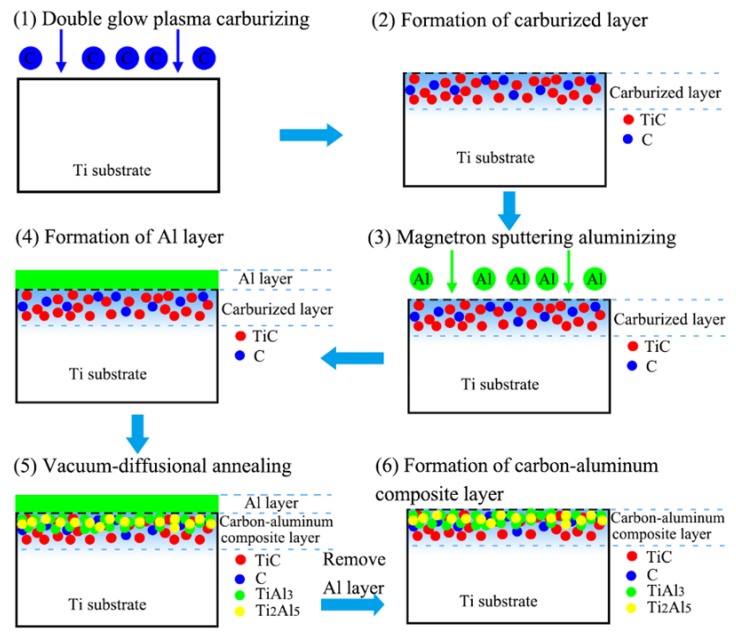
Schematic diagram of the formation process of the carbon–aluminum composite layer.

Figure 13 shows a schematic diagram of the formation process of the carbon–aluminum composite layer. The formation process mainly involves three parts: double glow plasma carburizing, magnetron sputtering aluminizing, and vacuum-diffusional annealing treatment. 

During the double glow plasma carburizing process, the active C atoms were adsorbed onto the surface of the titanium substrate and formed the concentration gradient of C, which resulted in the diffusion of C atoms from the surface to the inner substrate along the grain boundary and the crystal defects to form the solid solutions. At the same time, C reacted with Ti to form TiC, which was confirmed by the results of the XPS and XRD experiments in this work. 

During the magnetron sputtering process, the aluminum atoms were sputtered into the plasma field and collided with the electrons, at which point they were ionized into aluminum ions (Al^+^). Under the action of an electric field, Al^+^ rushed to the surface of the Ti substrate and deposited to form an Al layer after which the interdiffusion region was formed on the interface of Al/Ti.

During the vacuum-diffusional annealing process, the thickness of the diffusion layer that mainly contained the TiAl_3_ phase greatly increased. Under the action of concentration gradient forces, the Al atoms from the Al layer diffused toward the Ti substrate side continuously. When the concentration of Al atoms exceeded the equilibrium concentration in the Ti, the intermetallic compound of Ti–Al precipitated nucleation in the supersaturated solid solution of Ti(Al). Meanwhile, the decrease in the Al atoms’ concentration caused by the precipitation of the Ti–Al intermetallic compound accelerated the diffusion of Al atoms into the Ti side. This diffusion process led to the continuous formation of a supersaturated solid solution Ti(Al) and Ti–Al phases, thereby achieving the formation of Ti–Al phases in the diffusion layer on the Ti substrate. The carbon–aluminum composite layer can be formed composed of Ti–Al phases and TiC phases.

### 4.2. The Diffusion Layer and Composition

It is widely known that the dependence of annealing time on the thickness of the diffusion layer can be presented by the following equation [30,31]:

Y = kt^n^, (2)

ln k = 7.16 − 20473.7/T,(3)

where Y is the thickness of the compounds (m), t is the diffusion time (s), k is the rate constant, T is the diffusion temperature (K), and n is the kinetic exponent (in the present case, n = 0.5). When n is equal to 0.5, the growth of the reaction layer is controlled by parabolic growth; when n is 1, the growth of the reaction layer is dominated by linear growth. It was calculated that after vacuum-diffusional annealing at 550, 600, and 650 °C for 6 h, the thicknesses of the diffusion layers were about 2.9 μm, 12.4 μm, and 44 μm, which are larger than the average thickness of diffusion layers of about 2 μm, 5 µm, and 21 µm in this work. Thus, it can be inferred that the carburized layer acts as a barrier to retard the diffusion of Al atoms, and the thickness of the diffusion layer containing the phases of TiAl_3_ and Ti_2_Al_5_ increased slowly.

Sun et al. [32] studied the solid phase diffusion reaction behavior of Al/Ti and found that a solid solution of Ti(Al) had formed in the titanium layer. When aluminum is superfluous, it is easy to form the intermetallic compound, TiAl_3_. In this study, based on the XRD analysis, a small amount of the TiAl_3_ phase was formed (Figure 5, S2 and S3), indicating the diffusion reactions among the solid phases at 550 °C and 600 °C. With the increase in temperature, more TiAl_3_ and a small amount of Ti_2_Al_5_ phases were formed due to a further diffusion reaction between the solid titanium and aluminum. Guo et al. [33] worked on the diffusion of Ti/Al/Ti by hot pressing and found that the reaction layer consisted of TiAl_3_ and Ti_2_Al_5_ phases. The authors considered that the latent heat released by the diffusion process of the aluminum layer may have caused localized heating (up to 1100 °C), which led to the diffusion of aluminum and the generation of Ti_2_Al_5_ in the surrounding areas. This is consistent with the XRD analysis, as shown in Figure 5. According to the thermodynamic analysis of the Al–Ti–C system [34], it is clear that the TiC phase was more thermodynamically stable than the Ti–Al phases due to its lower △G. Therefore, during the formation of the diffusion layer, the TiC phase will be excised in the diffusion layer when the Ti–Al phases are formed. Based on the above analysis, an increase in the vacuum-diffusional annealing temperature promoted the diffusion of Al and the formation of TiAl_3_ and Ti_2_Al_5_.

### 4.3. Properties of the Carbon–Aluminum Composite Layer 

The results of the hardness and wear properties analysis indicate that the carbon–aluminum composite layer composed of Ti-Al phases and TiC phase can effectively reduce the friction coefficient of the pure titanium and improve its wear properties at room temperature. The excellent performance of sample S4 examined by indentation and ball-on-disc sliding tests can be explained by the formation of Ti-Al phases. It can be seen in Figure 5 that with increasing vacuum-diffusional annealing temperature, the intermetallic compounds of Ti-Al phases were noticeably increased, while the Ti phase decreased significantly. The micro-hardness of the Ti-Al phase can be considered higher than that of the Ti phase. Therefore, the Ti-Al phases in the carbon–aluminum composite layer seem to improve the hardness of the micro-area around the TiC phase. Due to the micro-area hardening caused by Ti-Al phases, the micro-area deformation resistance of the carbon–aluminum composite layer has been enhanced. This situation will be beneficial to demonstrate the high hardness characteristics of the TC phase. Accordingly, the hardness of the carbon–aluminum composite layer increased significantly. 

It is known that the factors that influence the friction coefficient include lubrication, surface roughness, temperature, load, and friction material [35]. According to the results of the friction coefficient in Figure 10, sample S4 still exhibits a low friction coefficient of about 0.33, compared with that of samples S2, S3, and TA2. Considering the observed micropores formed at the interface of Al/Ti (Figure 7d) during the vacuum-diffusional annealing process, this could be changing the surface roughness of sample S4 after the Al layer is removed. It is acceptable that the growth rate of hardness is significantly greater than the range of decline in the friction coefficient. Figure 12 shows the wear volumes of samples S1–S4 and TA2, which indicates that sample S4 had the best wear resistance due to high hardness and a low friction coefficient. 

## 5. Conclusions

The formation process and properties of the carbon–aluminum composite layer were investigated in the temperature range 550–650 °C for 6 h. The main conclusions are summarized as follows:

(1) The carbon–aluminum composite layer consisting of TiAl_3_, Ti_2_Al_5_, and TiC phases can be synthesized on the surface of pure titanium by double glow plasma carburizing, magnetron sputtering aluminizing, and vacuum-diffusional annealing treatment.

(2) With the vacuum-diffusional annealing temperature increasing, Al atoms diffused into the carburized Ti substrate to form a diffusion layer about 2 μm, 5 µm, and 21 µm at 550, 600, and 650 °C for 6 h. The thickness of the diffusion layer increased slowly related to the carburized layer which acts as a barrier to retard the diffusion of Al atoms.

(3) XRD and XPS analyses indicated that the carbon–aluminum composite layer consists of TiAl_3_, Ti_2_Al_5_, and TiC phases at 650 °C for 6h. The TiC phase formed by double glow plasma carburizing was excised in the composite layer when the Ti–Al phases were formed during the vacuum-diffusional annealing process.

(4) The hardness and wear resistance of the carbon–aluminum composite layer were significantly improved due to the formation of intermetallic compounds Ti–Al phases, which caused the micro-area hardening and enhanced the micro-area deformation resistance to exhibit a high hardness of the TiC phase in the carbon–aluminum composite layer. The maximum hardness of the samples reached 1231 HV_0.1_, the minimum friction coefficient of the samples was determined to be 0.33, and the minimum wear volume of the samples was calculated as 0.018 mm^3^.

According to the obtained results, the carbon–aluminum composite layer improves the wear resistance of the pure titanium while avoiding the problem of the adhesion of the coating-substrate in other surface treatment technologies. We argue that this strategy is applicable to wider applications for titanium and titanium alloy.

## Figures and Tables

**Table 1:** Processing parameters of double glow plasma carburizing.

Parameter	Value
Source voltage/V	800
Workpiece voltage/V	450
Ar pressure/Pa	30
Distance between workpiece and source/mm	20
Temperature/°C	900
Time/h	3

**Table 2:** Processing parameters of magnetron sputtering aluminizing.

Parameter	Value
Base pressure/Pa	9.0 × 10^−4^
Target Power/W	150
Ar flow rate/sccm	20
Work pressure/Pa	0.2
Time/h	3

**Table 3:** Processing parameters of the vacuum-diffusional annealing treatment.

Sample No.	Temperature (°C)	Degree of Vacuum (Pa)	Time (h)
S1	-	-	-
S2	550	4 × 10−3	6
S3	600	4 × 10−3	6
S4	650	4 × 10−3	6

**Table 4:** Chemical composition (at.%) in positions 1–5 as shown in Figure 8a–d, obtained from the energy dispersive X-ray spectrometer (EDS) analysis.

Sample	Element	Position				
		1	2	3	4	5
S1	Al	90.52	87.21	14.92	2.04	-
	Ti	/	2.24	68.45	78.32	81.38
	C	9.48	10.55	16.63	19.64	18.62
S2	Al	92.88	83.58	33.97	1.38	-
	Ti	/	8.99	53.19	78.35	84.39
	C	7.12	7.43	12.84	20.27	15.61
S3	Al	93.54	89.61	56.91	2.21	-
	Ti	/	3.13	24.45	80.2	83.86
	C	6.46	7.26	18.64	17.59	16.14
S4	Al	60.05	61.39	5.05	-	-
	Ti	20.53	21.89	79.00	85.03	85.97
	C	19.42	16.72	15.95	14.97	14.03

**Table 5:** Geometry parameters of the wear scar profile and the calculated wear volumes.

Sample	Wear Scar Depth (mm)	Wear Scar Width (mm)	Wear Volume (mm^3^)
TA2	0.015	1.054	0.220
S1	0.007	0.958	0.078
S2	0.005	0.725	0.023
S3	0.005	0.729	0.024
S4	0.003	0.792	0.018
